# Improved SARS-CoV-2 sequencing surveillance allows the identification of new variants and signatures in infected patients

**DOI:** 10.1186/s13073-022-01098-8

**Published:** 2022-08-12

**Authors:** Antonio Grimaldi, Francesco Panariello, Patrizia Annunziata, Teresa Giuliano, Michela Daniele, Biancamaria Pierri, Chiara Colantuono, Marcello Salvi, Valentina Bouché, Anna Manfredi, Maria Concetta Cuomo, Denise Di Concilio, Claudia Tiberio, Mariano Fiorenza, Giuseppe Portella, Ilaria Cimmino, Antonio Sorrentino, Giovanna Fusco, Maria Rosaria Granata, Pellegrino Cerino, Antonio Limone, Luigi Atripaldi, Andrea Ballabio, Davide Cacchiarelli

**Affiliations:** 1 Telethon Institute of Genetics and Medicine (TIGEM), Armenise/Harvard Laboratory of Integrative Genomics, Pozzuoli, Italy; 2 Next Generation Diagnostic srl, Pozzuoli, Italy; 3 Centro di Referenza Nazionale per l’analisi e studio di correlazione tra ambiente, animale e uomo. Istituto Zooprofilattico Sperimentale del Mezzogiorno, Portici, Italy; 4 UOC Microbiologia e Virologia, P.O. Cotugno A.O. dei Colli, Naples, Italy; 5 Department of Translational Medicine, University of Naples Federico II, Naples, Italy; 6 UOC Epidemiologia e Prevenzione, ASL Napoli 2 Nord, Dipartimento di Prevenzione, Casavatore, Italy; 7 Department of Molecular and Human Genetics, Baylor College of Medicine, Houston, TX USA; 8 Jan and Dan Duncan Neurological Research Institute, Texas Children Hospital, Houston, TX USA; 9 SSM School for Advanced Studies, University of Naples Federico II, Naples, Italy

## Abstract

**Background:**

Genomic surveillance of severe acute respiratory syndrome coronavirus 2 (SARS-CoV-2) is the only approach to rapidly monitor and tackle emerging variants of concern (VOC) of the COVID-19 pandemic. Such scrutiny is crucial to limit the spread of VOC that might escape the immune protection conferred by vaccination strategies or previous virus exposure. It is also becoming clear now that efficient genomic surveillance would require monitoring of the host gene expression to identify prognostic biomarkers of treatment efficacy and disease progression. Here we propose an integrative workflow to both generate thousands of SARS-CoV-2 genome sequences per week and analyze host gene expression upon infection.

**Methods:**

In this study we applied an integrated workflow for RNA extracted from nasal swabs to obtain in parallel the full genome of SARS-CoV-2 and transcriptome of host respiratory epithelium. The RNA extracted from each sample was reverse transcribed and the viral genome was specifically enriched through an amplicon-based approach. The very same RNA was then used for patient transcriptome analysis. Samples were collected in the Campania region, Italy, for viral genome sequencing. Patient transcriptome analysis was performed on about 700 samples divided into two cohorts of patients, depending on the viral variant detected (B.1 or *delta*).

**Results:**

We sequenced over 20,000 viral genomes since the beginning of the pandemic, producing the highest number of sequences in Italy. We thus reconstructed the pandemic dynamics in the regional territory from March 2020 to December 2021. In addition, we have matured and applied novel proof-of-principle approaches to prioritize possible gain-of-function mutations by leveraging patients’ metadata and isolated patient-specific signatures of SARS-CoV-2 infection. This allowed us to (i) identify three new viral variants that specifically originated in the Campania region, (ii) map SARS-CoV-2 intrahost variability during long-term infections and in one case identify an increase in the number of mutations in the viral genome, and (iii) identify host gene expression signatures correlated with viral load in upper respiratory ways.

**Conclusion:**

In conclusion, we have successfully generated an optimized and cost-effective strategy to monitor SARS-CoV-2 genetic variability, without the need of automation. Thus, our approach is suitable for any lab with a benchtop sequencer and a limited budget, allowing an integrated genomic surveillance on premises. Finally, we have also identified a gene expression signature defining SARS-CoV-2 infection in real-world patients’ upper respiratory ways.

**Supplementary Information:**

The online version contains supplementary material available at 10.1186/s13073-022-01098-8.

## Background

Severe acute respiratory syndrome coronavirus 2 (SARS-CoV-2), the etiological agent of the coronavirus disease 2019 pandemic (COVID-19), is a positive-sense, single-stranded RNA virus belonging to the genus *Betacoronavirus* [1]. The spreading of COVID-19 cases around the globe promoted the natural selection of viral variants harboring gain-of-function mutations: in 2020 the substitution of aspartate-614 with glycine in the Spike protein of the original Wuhan strain (Spike D614G) guaranteed the first major competitive advantage, both in terms of replication efficiency and infectivity [2]. Consequently, it should not come as a surprise that while being marginally prevalent at the beginning of the pandemic, the frequency of the D614G mutation rapidly increased in Europe starting from February 2020. In the following year, this mutation characterized 99% of SARS-CoV-2 sample incidence [3]. The virus acquired further mutations since the beginning of the pandemic, increasing its genetic diversity, consequently leading to the spread of several viral lineages (variants), each characterized by a specific set of mutations [4]. Some variants’ mutations granted particular advantages to the viral spreading, thus leading to their domination over the population of origin [5–7]. These are usually characterized by enhanced infectivity and transmissibility, hence the World Health Organization (WHO) defines such as variants of concern (VOC) [8]. Although VOC are still responsive to current treatments as the ancestral virus does [9], some standalone SNPs such as the Spike mutation E484K, cause a lower sensitivity to both monoclonal antibodies and vaccine-induced human serum [10, 11]. This accentuates the importance of tracking not only viral infections but also the viral variants causing them. Such an effort is resolutely imperative now as new selective stimuli, such as vaccines and antibody therapies, are being introduced in the general population.

Along with genotyping of the SARS-CoV-2 genome, it is becoming pertinent to acquire insights into the cellular response of the host cells as an approach to monitor disease progression, patient stratification and biomarkers identification. Now more than ever, providing simple and cost-effective tools to comprehensively profile virus genome and host transcriptome became imperative to effectively tracking and isolating focal areas of variants eluding vaccination. At the moment, the vast majority of studies concerning the elucidation of the molecular bases of viral infection have been carried out in in vitro virus-infected models (e.g., cell lines, organoids) [12–14]. While these approaches guarantee an easy-to-handle and rapid solution, they lack generalization, as they are narrowed to the system used, and do not account for the physiological interaction between host infected cells and their microenvironment (e.g., immune cells). However, due to the scarce and exiguous quality of nasal swab RNA, combined with the excessive costs of sampling a considerable cohort of COVID-19 positive patients, it has been challenging so far to obtain consistent and cost-effective patient gene expression data.

In our work we aimed at filling these gaps by creating an integrated genomic workflow that allows, from the diagnostic extract, to reconstruct the SARS-CoV-2 complete genome using a customized amplicon-based method, and to retrieve global gene expression of the host airway epithelium, via an adapted RNA-seq approach. As further discussed, such an affordable and scalable workflow can be implemented in laboratories lacking automation and equipped with benchtop sequencers. Nevertheless, we applied such workflow on genome-scale NGS sequencers to perform an efficient genomic surveillance in the South of Italy. Our effort allowed us to establish novel procedures to prioritize new emerging variants and to identify molecular signatures associated with a viral infection, maturing a powerful tool for disease prevention, diagnosis, and, potentially, personalized treatment.

## Methods

### Samples collection, RNA extraction, and SARS-CoV-2 testing

Sample handling, diagnostics, and logistics were carried out by Ospedale Cotugno as regional reference center for infectious diseases and Istituto Zooprofilattico Sperimentale del Mezzogiorno (IZSM), as coordinator of Coronet network of Regione Campania. All samples were randomly collected in Campania, Italy, as part of the institute’s and local health services diagnostic activity during 2020 and 2021. In most of the cases, after a first diagnosis, a second RNA extraction and qPCR were performed, by IZSM to generate uniform qPCR results. RNA extraction was performed in general by using either the Maelstrom 9600 (TANBead), GeneQuality X120 (AbAnalitica), or Abbott m2000sp automatic platforms according to the manufacturer’s specifications. SARS-CoV-2 abundance in each sample was tested by using either the Allplex 2019-nCoV Assay (Seegene), Real Quality RQ-2019-nCoV kit (AbAnalitica), or Abbott RealTime SARS-CoV-2 Amplification Kit by detecting at least two of the N, E or RdRP, SARS-CoV-2 genes. In all analyses where the Ct value of each subject was employed, the average Ct of the three genes was calculated and used. A total of about 22228 were used for SARS-CoV-2 whole genome sequencing. Out of these, 387 samples were used to investigate host gene expression and were divided into two cohorts depending on the viral variant identified: the first cohort included 162 samples assigned to the B.1.x variant and the second included 225 samples assigned to the *delta* avariant. In addition, 300 RNA extracts from SARS-CoV-2 negative swabs were also sequenced.

### SARS-CoV-2 WGS and computational analysis

All procedures including library preps were performed with standard filtered low-retention tips and each step of the library preps was performed in a separate PCR hood, located in different rooms with dedicated pipettes and thermocyclers. Prior and after each step, decontamination occurred by using a combination of UV irradiation, 0.5% bleach, and DNAzap (Thermofisher). Libraries were always prepared in multiples of 96 samples arrayed in a 96-well plate and at least 5 blank samples (water) were added in each plate to monitor cross-contamination. Library generation for SARS-CoV-2 genome sequencing was performed by using a modified and optimized version of the amplicon-based ATOPlex RNA Library Prep kit (MGI Tech) starting from 5, 2.5, and 1.25 μL of unquantified extracted RNA. The volume of reagents was reduced to 1/2, 1/4, and 1/8 of the originally recommended volumes, respectively. The sequencing strategy was also optimized to increase the sequencing throughput from 96 libraries per run to 384 by manually loading the 4 flow-cell lanes of PE100 cycles 320G flow-cell (MGI Tech). Further multiplexing can be achieved by increasing the indexing up to 768 libraries per run, as shown by randomly subsampling 1.25 million reads per sample. Similarly, two 96 library pools can be sequenced on two lanes of a PE100 cycles SP/S1 Novaseq flow-cell (Illumina). The two sequencing technologies show comparable performances [15].

One-step tests were performed by merging the 1st and 2nd PCR step of the ATOPlex RNA Library Prep kit. In particular, we prepared a PCR reaction mixture containing all the components of the 1st PCR step plus the “PCR Primer block” and the “PCR additive” of the 2nd PCR. The PCR was then conducted using the program suggested in the original protocol [16]. However, to decrease the amount of unincorporated primers at the end of the amplification, the number of cycles was increased from 13 to 25. For the same reason, the concentration of the “PCR Primer Pool” component was decreased to 1/75 of the original one. As soon as the reaction cooled down to 4 °C, the indexing primers were added and the reaction was allowed to continue for further 15 cycles. All the reagents, except the PCR Primer Pool” were used at the same concentration as suggested by the original user manual [16].

FASTQ files generated by the MGI sequencer (DNBSEQ-G400) were used as input for the pre-processing pipeline. The pipeline used was adapted from MGI-tech-bioinformatics [17] and a threshold coverage of at least 30X was used to call each base in the consensus sequence. It was further parallelized and automated to process 100 samples/h using Nextflow [18]. SARS-CoV-2 viral load was implied as the percentage of reads aligning to the viral genome with respect to the co-amplified Lambda phage genome added as spike-in at the beginning of the library preparation. A co-amplified host GAPDH locus was used in the pipeline for internal positive control. Only samples with a minimum SARS-CoV-2/Lambda reads ratio of 10%, 50,000 SARS-CoV-2 reads and at least 50% of genotyped bases were considered for GISAID submission. Blank samples always displayed around 1% SARS-CoV-2/Lambda reads ratio and almost never exceeded 10%. Upon GISAID submission, only samples uploaded before 2021-05-26, labeled as complete by GISAID and with >95% of genotyped bases were used for further analysis. Furthermore, to normalize sequencing statistics when comparing the three solutions developed, only samples with Ct values lower than or equal to 33 were selected.

The phylogenetic analysis was generated using Nextstrain [19] standard pipeline on a random subsample of sequences generated until 2022-03-30. Tree visualization was performed using R (v. 4.1.0) with the packages ape (v. 5.5), ggTree (v. 3.0.2), phangorn (v. 2.7.1), and castor (v. 1.6.8). BA.1.21.1 tree was generated by using the *omicron* sequences produced and a random sample of sequences from GISAID assigned to other lineages (GISAID epi set: EPI_SET_20220509ow).

The frequency of mutations of concern was analyzed by considering a mutation as “expected” if its frequency in a certain lineage was higher than 30% over the total number of worldwide samples assigned to that specific lineage. Mutation trends clustering was performed using the PAM clustering method as follows. The input for the algorithm was a mutation × month matrix indicating the frequency of each mutation in each month. Out of all the mutations detected, only the ones reaching 5% of incidence at least once during the period of analysis were used for clustering. The number of clusters was chosen by using the silhouette method (factoextra v. 1.0.7). This yielded to 3 optimal clusters. A further round of clustering on the first two clusters (*k*=28 and *k*=3, chosen with the silhouette method) resulted in a total of 32 groups. The same number of clusters was chosen for both the analysis performed in May 2021 and January 2022. Finally, clusters too similar were manually merged.

### Host mRNA-seq and computational analysis

RNA-seq was performed by using the 3'DGE mRNA-seq clinical grade sequencing service (Next Generation Diagnostic srl) [20] which included library preparation, quality assessment and sequencing on a NovaSeq 6000 sequencing system using a single-end, 100 cycle strategy (Illumina Inc.). Prior to library preparation, a 40–60 μL unquantified swab RNA extract (1–5 ng/μl estimate) was treated with DNAse I (Life Technologies), purified, and concentrated to a final volume of 5μL, all volume was then used in the library preparation reaction. One or two sets of 96 library pools were sequenced on a SE100 cycles SP Novaseq flow-cell (Illumina).

Illumina NovaSeq raw data were initially analyzed by Next Generation Diagnostic srl proprietary 3'DGE mRNA-seq pipeline (v2.0) which involves a cleaning step by quality filtering and trimming, alignment to the reference genome, and counting by gene [21–23].

Samples were considered qualitatively sufficient and retained based on the number of detected genes (≥ 5000) and the percentage of reads assigned to genes ( ≥ 20%). Data were normalized via the *cpm* function from the edgeR [24] package (v. 3.34.1). Principal component analysis was conducted by *prcomp* function from R (v. 4.2) on normalized, log-transformed counts.

Correlation analysis between Ct values and gene expression was performed on genes that were expressed (i.e., CPM > 1) in at least 70% of the entire dataset (8100 genes for B.1 and 5525 for Delta). The test was performed using the function *cor.test* from R (v. 4.2). Anti-correlation was defined for results with *p*-value < 10^-4^. Pathway and gene sets enrichment analysis was conducted using the enrichR [25–27] package (v. 3.34.1).

## Results

### A systematic approach allows the generation of large and robust genomic data in a cost-effective manner

**Fig. 1:**
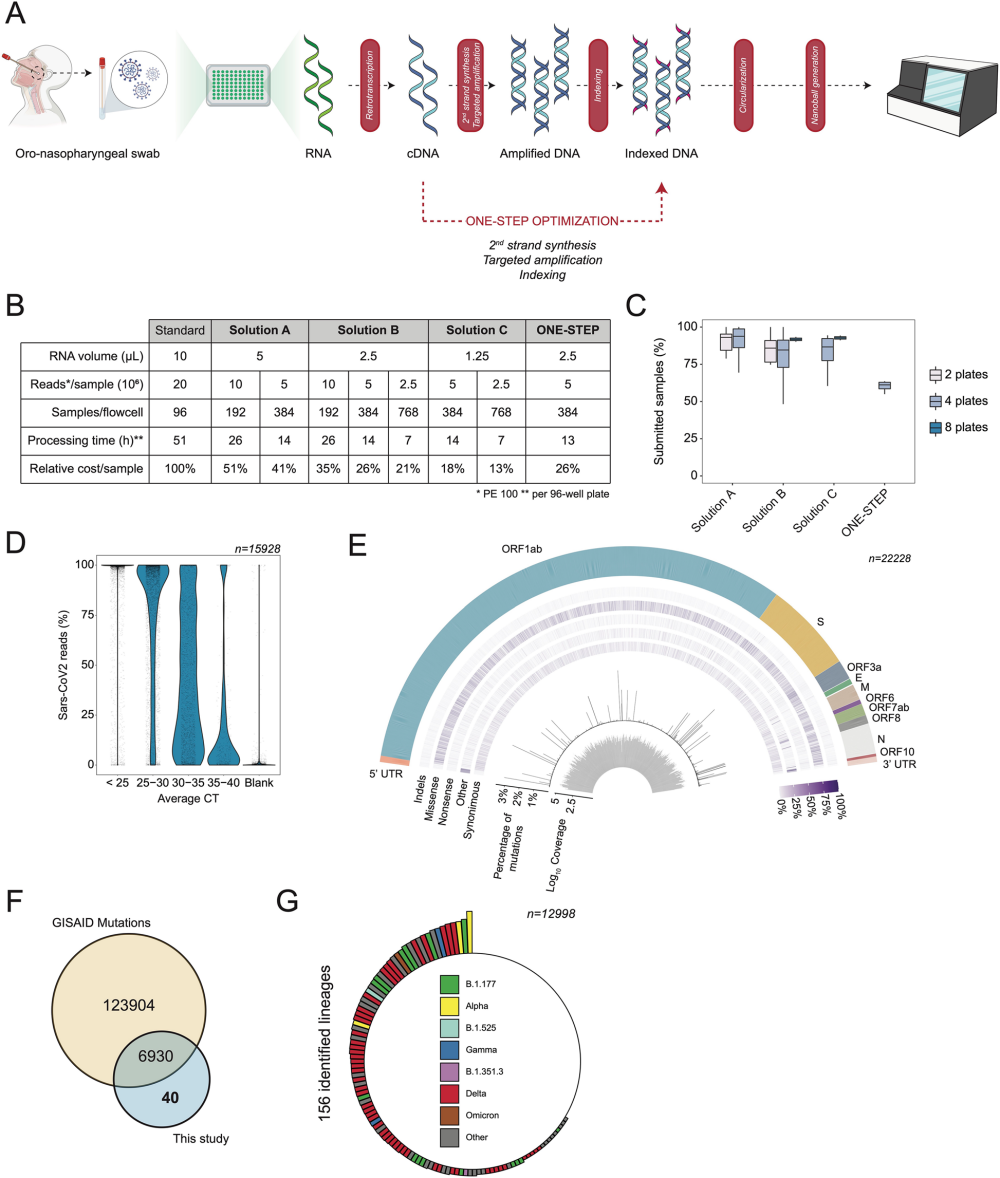
A systematic approach allows the generation of large and robust genomic data in a cost-effective manner. **A** Schematic representation of the workflow set up to collect, process, and analyze a considerable number of viral genomes. Top: Oronasopharyngeal swabs are performed to diagnose the presence of the SARS-CoV-2 genome in patients and extract its RNA. Subsequently, viral RNA is retrotranscribed and subjected to two PCR steps to amplify and index the obtained cDNA. After circularization and nanoball generation, the obtained library is then sequenced and analyzed. Bottom: As an alternative and faster approach, an optimized approach enables the amplification and indexing to occur in one PCR step. **B** Multiple solutions were tested to optimize the workflow. The table reports the input RNA volume, the amount of reads produced per sample, the number of samples loaded per flowcell, the average time required to process a 96-well plate, and the relative cost per sample. Cost details are reported in Additional file 2: table S1. **C** Boxplot showing the percentage of samples submitted on the GISAID platform, divided by each tested solution. Only samples with an average Ct value < 33 were considered. **D** Violin plot showing the distribution of the percentage of SARS-CoV-2 reads detected for different ranges of CTs. n:sample size. **E** Variant annotation, cumulative frequency, and sequencing coverage of each position of the SARS-CoV-2 genome. **F** Venn diagram showing the intersection between mutations detected in all the sequenced genomes worldwide (yellow) and the mutations found in this study (light blue). **G** Representation of all the 156 lineages identified in this study. The length of the bars is indicative of the number of samples for each lineage in the logarithmic scale. Colored bars indicate VOC

Besides screening and diagnosis, one of the major needs related to the SARS-CoV-2 pandemic is to collect and analyze a considerable amount of viral genomes, to guarantee a rapid geographical and continuous surveillance of VOC. To achieve this goal, we developed a systematic workflow that allows the collection, whole genome sequencing (WGS), cloud data processing, and sharing of up to 4500 SARS-CoV-2 genomes per week. Our approach is based on the optimization of an amplicon-based workflow [16] (see the “Methods” section) (Fig. 1A). To both increase processivity and efficiently reduce costs, the protocol was tested and validated with a decreasing amount of input RNA for the generation of the libraries. In particular, we tested 5 μL, 2,5 μL, 1,25 μL of unquantified RNA and proportionally scaled down the reaction volumes to 1/2, 1/4, and 1/8 (solution A, B, and C, respectively) (Fig. 1B, C, Additional file 1: Fig. S1A and Additional file 2: Table S1). Neither the number of mapping reads, the genome coverage, nor the number of sequences passing our quality filters and submitted to GISAID were significantly affected by volume reductions.

Being able to rapidly process the RNA sample to the final viral genome consensus is a critical aspect for retrieving meaningful data on the SARS-CoV-2 genome surveillance in a territory. We addressed this point by both optimizing the steps required for library generation and adjusting the number of samples sequenced in each run (Fig. 1B). Particularly, we merged the targeted and the indexing amplification steps in a single PCR (Fig. 1A, see the “Methods” section). Such a one-step strategy has an efficiency of ~ 60%; however, it allows to remove a magnetic beads purification step and thus reducing the hands-on time for library generation by ~40% (Fig. 1B). In parallel, sequencing flow performance and efficacy were tested and validated against increasing number of samples per run, as such can have an adverse effect on the quality of the resulting viral sequences. We compared QC statistics obtained by sequencing at a depth of ~ 9, 4.5, and 2.75 million 100bp paired-end reads per sample. This translates into sequencing two (192 samples), four (384 samples) or eight (768 samples) 96-well plates per run. As expected, the total amount of reads decreases by increasing the number of samples per flow-cell; nevertheless, the downstream parameters were not affected, allowing us to genotype a similar amount of SARS-CoV-2 genomes (i.e., GISAID database sequence acceptance rate—Fig. 1C).

**Fig. 2:**
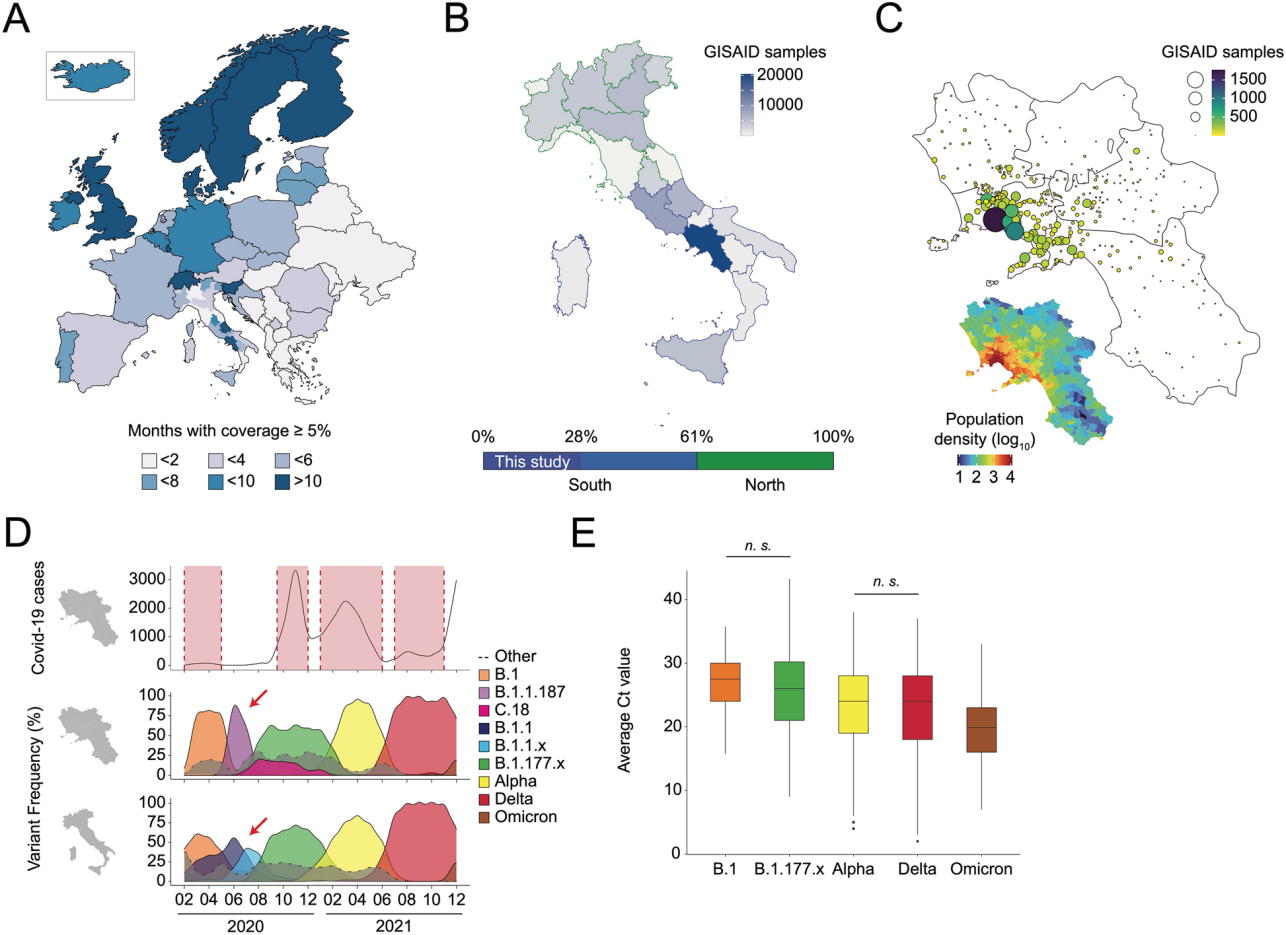
Characterization of SARS-CoV-2 genome evolution in the south of Italy. **A** Geographic map representing European States, colored by the number of 2021 months with at least 5% of viral genomes sequenced, compared to new cases. Only for Italy, individual regions are displayed. **B** Top: geographic map representing Italian regions, colored by the number of genomes deposited on the GISAID platform. Bottom: percentage of genomes deposited on GISAID over the total Italian sequences, divided in Northern (green) and Southern (blue) regions. 28% of the overall Italian sequences have been produced by this study (dark blue). **C** Geographic distribution in Campania of the genomes analyzed in this study (top) relative to the population density (bottom). **D** Density plots showing the distribution, in time, of the most frequent variants described in this study (middle) or in Italy (bottom) relative to the Campania infection curve (top) and waves (red-colored areas). Red arrows highlight different variants dynamics between regional and national level, in a certain period of time. **E** Distribution of the average CT value across different Variants of Concern (VOC). Only not significant (n.s.) pairwise comparisons are reported (Bonferroni adjusted *p*-value > 0.05)

Therefore, by finely tuning the starting amount of RNA, the library generation steps, and the number of samples loaded in each sequencing run, we were able to decrease both the processing time and the costs. Using our optimized SARS-CoV-2 WGS workflow solution B, during 2021, we were able to process, as a proof-of-principle approach, about 30,000 swabs and sequence 22,228 of them, 17,193 of which generated high-quality genomes (defined as those complete genomes with a percentage of Ns lower than 5%, Additional file 2: Table S2). A strong correlation between the number of reads detected in each sample and the Ct values obtained from a diagnostic qPCR was observed (Fig. 1D). SARS-CoV-2 reads showed a proportional rate with respect to Ct in the intervals between 40 and 25 Ct while reaching saturation <25 Ct. Altogether, these observations suggest that our WGS approach reliably quantifies the viral load and provides us with crucial metadata to correlate higher virus titer to specific virus lineages and a transcriptional response from host cells (see later in Figs. 2 and 4). The robustness of this approach was further established by analyzing the mean coverage level, which appeared to be homogeneous across the entire sequence (Fig. 1E). This piece of evidence confirmed the absence of major biases in the single nucleotide evaluation: hence, we investigated the SNPs information derived from our genomic screening and determined missense and synonymous mutations to be the most frequent across the entire genome, although few positions appeared to be more prone to mutate (Fig. 1E). Indeed, a number of mutations (Additional file 2: Table S3) were efficiently detected in our dataset, 194 of which were previously unknown (Additional file 2: Table S4) and 40 only identified in Campania (Fig. 1F). Interestingly, out of the 194 mutations first collected in the region, 20 fall within the Spike gene, at or nearby residues reported to be relevant for neutralizing antibody binding [28]. Taken altogether the mutations detected allowed us to identify 156 different SARS-CoV-2 pangolin [6] lineages (Fig. 1G), some of which were retrieved for the first time thanks to our activity (see below).

### Characterization of SARS-CoV-2 genome evolution in the south of Italy

As aforementioned, starting from the end of December 2020 to the first week of 2022, we have sequenced, uploaded to GISAID, and analyzed 17,193 SARS-CoV-2 genomes. Our workflow was tested throughout the Campania region, which includes the major southern Italian metropolitan areas and some of the most densely inhabited cities in Europe. Globally, we were able to sequence, in most months during 2021, at least 5% of all COVID-19 positive samples (Fig. 2A), making Campania compliant with EC/ECDC recommendations, reaching a sequencing coverage comparable to that of North-European countries. In fact, our dataset represents almost half of all sequences retrieved and uploaded from the south of Italy and 28% of all sequences produced in the country (Fig. 2B). Samples were collected starting from March 2020, by randomly selecting positive swabs and reflecting population demographics of sex, age, and the geographical distribution across the area of interest (Additional file 1: Fig. S2A and Fig. 2C).

The analysis of samples collected during the whole pandemic period allowed us to unveil the full dynamics of the SARS-CoV-2 outbreak in Campania. We indeed reconstructed the distribution of all the VOC arrived in Campania (Additional file 1: Fig. S2B), particularly the *delta* (represented by B.1.617.2 and AY.* lineages) and *alpha* (B.1.1.7 and Q.*) VOC, which represented the vast majority of variants detected (71.6%). In accordance with worldwide data, the first VOC arriving in the region, starting from December 2020, were the B.1.1.7 and P.1 (*gamma* variant). Next, other VOC were detected in the region, including B.1.351, P.1, and B.1.1.529 lineages (i.e., *beta*, *gamma*, and *omicron* VOC, respectively). We also identified three main Variants of Interest (VOI); the B.1.427, B.1.525, and B.1.621 lineages (i.e., *epsilon*, *eta*, *mu*).

Out of the 156 viral lineages identified in the region, 5 were first recorded in Campania territory, namely B.1.1.187, B.1.177.33, B.1.177.75, C.18, and P.1.1 (Additional file 2: Table S5). In particular, the C.18 viral variant was first collected in July 2020, whereas its first record outside Campania was registered 3 months later, suggesting a possible epidemiological origin from our territory of investigation. Similarly, over 82% of B.1.1.187 samples collected during the pandemics were derived from Italy, all from Campania. Our analysis also showed that the first *gamma* VOC sub-variant identified (pangolin lineage P.1.1) was first sampled in Campania by our activity (Additional file 2: Table S6) and that it was specifically enriched in Italy, with sequences from Campania representing about 20% of all P.1.1 samples identified.

Looking at the whole picture, we determined that the main infection peaks in the region were associated with the spread of specific viral lineages (Fig. 2D top and middle overlays). Indeed, while the first wave of infections was mostly due to the ancestral B.1 lineage, the second one (autumn 2020) was led by the B.1.177 lineage (also referred to as the European or Spanish variant) and its sub-lineages. Interestingly, the time window between the first two infection peaks was characterized by two of the aforementioned lineages to be firstly detected in Campania; B.1.1.187 and C.18, associated with most of the COVID-19 cases during late spring and summer of 2020. These two variants distinguish the pandemics in Campania relative to the rest of Italy (Fig. 2D lower level, red arrow). In the same period in the rest of the country, infections were predominantly associated with other B.1 sub-lineages, mainly including B.1.1, B.1.1.305, and B.1.1.229.

Finally, the two infection peaks of 2021 were due to the spread of *alpha* and *delta* variants. These two VOC succeeded one after the other and accounted for almost all COVID-19 cases in the first (*alpha*) and second (*delta*) half of 2021. Interestingly, from December 2021 B.1.1.529 (*omicron*) variants started to emerge.

Since during this succession of variants in the regional territory, none of them ever reappears after being undermined by the subsequent one, it is fair to suppose that each variant has been substituted by one with higher fitness and capability to spread. To test this hypothesis, we looked at the viral loads in the upper respiratory ways of patients infected by the predominant variants in Campania (Fig. 2E). We observed a clear trend towards an increase of viral titer in patients during the pandemics, with a Ct value difference between *omicron* and the ancestral B.1 variant of −7.8 (*q* value < 2×10^−16^ pairwise Mann-Whitney test). A similar trend towards decreasing Ct values was observed also when taking into account all the variants identified in the region (Mann-Kendall test, p value=8.99×10^-10^, Additional file 1: Fig. S2C).

### High-throughput genomic surveillance allows the identification of new variants based on the analysis of single mutations

As, the comparative analysis of our dataset with GISAID world data allowed us to retrospectively identify viral variants firstly sampled in Campania (B.1.1.187 and C.18), we were interested to explore whether it was possible to unveil new viral lineages circulating in the territory. To achieve this goal, we explored several approaches. We mainly focused on the concept that a new SARS-CoV-2 variant is characterized by a specific set of mutations, therefore we generated approaches based on (1) mutations associated with a higher infectivity found in unexpected variants; (2) an increasing incidence of a set of mutations in a short time window; (3) the appearance of new mutations in samples collected by patients with persistent infections.

**Fig. 3:**
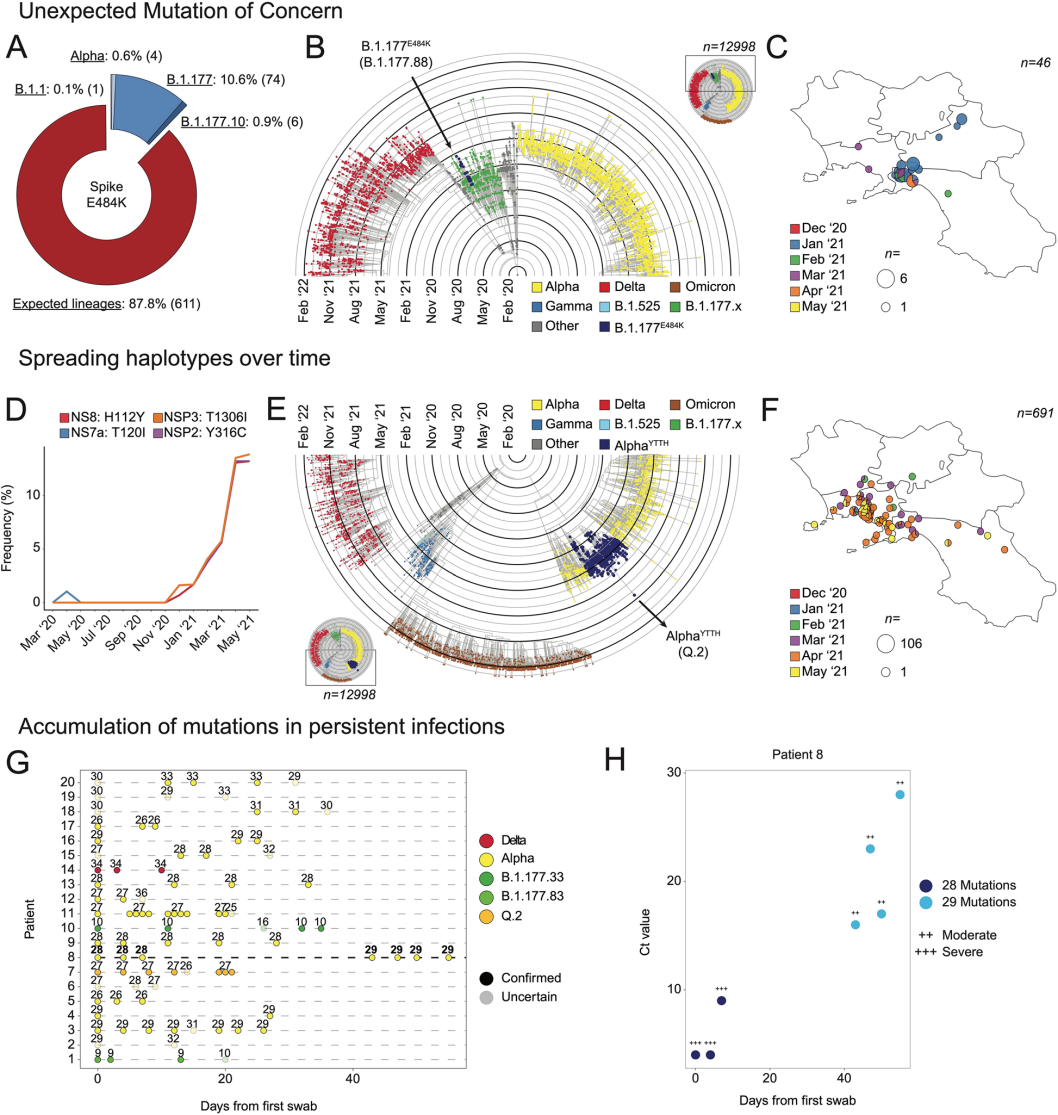
High-Throughput genomic surveillance allows the identification of new SARS-CoV-2 lineages. **A** Donut chart representing the amount of analyzed genomes presenting the Spike E484K mutation, divided by lineage. The definition of Expected lineage is described in the Methods. **B** Section of the phylogenetic tree representation of the whole dataset (*n*=12,998), colored by lineages. The identified lineage is reported (blue dots, left) and zoomed in (right). n:sample size. **C** Geographic distribution of genomic variants belonging to the identified lineage, colored by the collection date. The size of each pie chart is proportional to the number of samples in each geographic position. n:sample size. **D** Line plot showing the frequency trend of the selected mutations in time. **E** Section of the phylogenetic tree representation of the whole dataset (*n*=12,998), colored by lineages. The identified lineage is reported (arrow, blue dots). n:sample size. **F** Geographic distribution of genomic variants belonging to the identified lineage, colored by the collection date. The size of each pie chart is proportional to the number of samples in each geographic position. n:sample size. **G** Genomic characterization of twenty patients with long COVID-19 infection. The number of detected mutations is reported as a function of the number of days from the first swab. The assigned lineage (colors) and consistency (transparency) are also displayed. **H** Patient 8 genomic characterization relative to the number of detected mutations (colors), the infection load (*y*-axis), and symptoms severity (+++: severe; ++: moderate)

First, we explored SARS-CoV-2 “mutations of concern” genotyped in unexpected lineages (Fig. 3A and Additional file 1: Fig. S3A). Interestingly, we found that the Spike E484K substitution had an unexpected distribution in the lineage identified at the beginning of 2021. This mutation is typically found in P.1.x and B.1.351.x viral lineages and has been associated with a decreased sensibility to both monoclonal and BNT162b2 vaccine-induced antibodies [7, 10, 11, 29]. However, as of May 2021, ~21% carrying this mutation were associated with the B.1.177.x lineage. To further investigate this finding, we performed a phylogenetic analysis over our entire dataset using Nextstrain [19] and found that all B.1.177.x samples carrying the Spike E484K substitution (B.1.177^E484K^ samples) clustered in a specific and monophyletic clade branching within the B.1.177.x lineage (Fig. 3B). We further confirmed this finding by looking at the distribution of B.1.177^E484K^ samples in the phylogenetic tree containing all high-quality SARS-CoV-2 genomes from GISAID [6, 30]. This data points to the fact that B.1.177^E484K^ samples cluster in a monophyletic clade with an extremely high (0.99) support value, thus confirming regional level incidence (Additional file 1: Fig. S3B). Additionally, as the GISAID database revealed that B.1.177^E484K^ samples had been identified for the first time in Campania through our program, we investigated their geographic distribution in the regional territory to trace the epidemiological link (Fig. 3C). Surprisingly, these samples originated from a specific area between Naples and Salerno called “Agro Nocerino-Sarnese.” Combining these results, we hypothesized that B.1.177^E484K^ variant had probably arisen in this area in December (treetime divergence inferred interval: 2020-11-22~2020-12-21) and, then, spread nearby in Campania and in other confining Italian regions (mainly Lazio and Basilicata). Altogether, these observations allowed us to define a new SARS-CoV-2 lineage, which is now recognized by the Pangolin nomenclature B.1.177.88.

To identify any new variant rapidly growing in the territory as soon as it appears, we exploited another approach based on the incidence over time of each of the 6441 amino acid mutations we identified. As, by definition, a viral variant is defined by a specific combination of mutations, we looked at mutations that displayed similar trends in the same period and grouped them in clusters. In order to identify any potential new *alpha* subvariant growing in Campania, we applied this methodology to the data collected until may 2021, when the variant reached its maximum (Additional file 1: Fig. S3C). Several clusters clearly reflected the trends of known lineages, confirming the robustness of our approach; for instance, cluster 6 consisted of those substitutions that characterize the B.1.177.x lineage (namely N A220V and Spike A222V) and presented the same trend over time. Similarly, cluster 29 reflected the trend of B.1.1.7 lineage. Among these, cluster 18 was particularly interesting (Fig. 3D). It consisted of 4 mutations (NSP2 Y316C, NSP3_T1306I, NS7a T120I, NS8 H112Y) with the exact same frequency behavior over time, thus suggesting a possible SARS-CoV-2 haplotype. A further investigation revealed that these SNPs define a set of samples assigned to the B.1.1.7 lineage and specifically localized in Campania in May 2021 (B.1.1.7^YTTH^ samples). Similarly to the previous analysis, we carried out a phylogenetic analysis that confirmed B.1.1.7^YTTH^ as monophyletic (Fig. 3E and Additional file 1: Fig. S3D). While B.1.1.7^YTTH^ genomes did not show any geographic enrichment, its temporal distribution was indicative of an inland origin (Treetime divergence inferred interval: 2020-12-01~2020-12-06), followed by its spread first to the Neapolitan coast and then towards the Southern Neapolitan province (Fig. 3F). B.1.1.7^YTTH^ variant has been recognized, upon our alert, as one of the first B.1.1.7 sublineage by the Pangolin system and is now referred to as the Q.2 lineage.

By applying the same approach to the data produced till January 2022, we also identified a new *Omicron* subvariant (BA.1.21.1, Cluster 17 in Additional file 1: Fig. S3E and F). The variant is characterized by an early STOP codon mutation in NS7b (E3stop) and a SNP in Nsp12 (L749M). This variant was first collected at the end of 2021 and rapidly spread in Campania at the beginning during 2022, accounting for over 10% of all the infections in the region between January and March 2022.

Several reports [31] showed the accumulation of mutations in the SARS-CoV-2 genome during persistent infections. However, the frequency of such events is still overlooked. In order to possibly address this question and identify potential new variants, we analyzed swabs collected from 20 patients multiple times for over 40 days during prolonged infections (Fig. 3G). Age and immunological status highly varied across the patients (Table 1): patients’ age ranged from 13 to 88 (average 62) years and while most of them were affected by simple or bilateral pneumonia, six suffered a more severe respiratory failure and only one showed no COVID-related symptomatology. It is worth noting that, although most samples were collected during 2021, none of the patients had completed a three-dose SARS-CoV-2 vaccination cycle, 4 had only one vaccine dose, and most had no vaccination at all (13/20).

**Table 1:** Detailed clinical status of patients from Fig. 3G

Patient	Immune compromised	Main clinical symptoms	Comorbidities	Age	Vaccine	Outcome
1	Yes	Pneumonia	LNH	64	None	Healed
2	No	Pneumonia	Hemoperitoneum, anemia	30	None	Deceased
3	Yes	Respiratory failure	Pulmonary hypertension, NHL	64	Pfizer (×2)	N/A
4	No	ARDS	Diabetes, hypertension, ischemic heart disease	76	None	Healed
5	No	Mild respiratory failure	Necrotizing-hemorrhagic pancreatitis	60	None	Deceased
6	No	ARDS	Hypertension, dyslipidemia	61	None	Deceased
7	No	Bilateral pneumonia, fever, asthenia, myalgia, dyspnea	T2D, obesity, hypertension	59	None	Healed
8	No	Not specified severe symptomatology	Atrial fibrillation, T2D	78	None	Deceased
9	Yes	ARDS	Anemia, ALS, COPD	73	Pfizer (×1)	Healed
10	No	Respiratory failure	ARDS, sepsis, anemia, pulmonary hypertension	64	None	Healed
11	No	Bilateral pneumonia	None	88	None	Deceased
12	No	Bilateral pneumonia, fever, asthenia, myalgia, dyspnea	Hypertension, T2D, HCV, dyslipidemia, obesity	68	None	Healed
13	Yes	Pneumonia	NHL	73	Pfizer (×1)	Healed
14		N/A		87	N/A	Healed
15	No	respiratory failure	Psoriasis	44	None	Heled
16	No	Pneumonia, dyspnea, chest pain	Hypothyroidism, severe obesity	71	Pfizer (×1)	Healed
17	N/A	N/A	N/A	26	N/A	Healed
18	Secondary to chemotherapy	Asymptomatic	Ewing sarcoma	13	None	Healed
19	No	Fever, cough, dyspnea, pneumonia	Mixed dyslipidemia, obesity, hyperthyroidism, hypovitaminosis D	64	None	Healed
20	Yes	Pneumonia	Thymoma, Good’s syndrome	60	Pfizer (×1)	Healed

Sequencing of the viral genetic material confirmed no shift from a viral variant to another over time but each had a set of patient-specific mutations. However, looking at the individual mutations, in one patient (#8) there was an actual increase in the number of amino acid substitutions, as confirmed by several independent sequencing runs on several subsequent timepoints. The acquisition of the mutation (NSP13 R339C) was recorded only after 40 days from the first swab and did not correlate with an increase in the viral load or a worsening of the symptoms (Fig. 3H).

These results suggest that in specific conditions, such as over 40 days of persistent infection, the SARS-CoV-2 genetic consensus sequence can actually change, although the rate of such an event, as well as its biological significance, are not known yet.

Tracking new variants based on mutations arising in specific conditions is a novel approach for SARS-COV-2 surveillance. Here we showed that, by combining this approach with deep profiling of viral variability, new SARS-CoV-2 variants can be unveiled, even at the regional level.

### Transcriptional profiling of Sars-CoV-2-infected patients reveals a gene signature correlated with viral load and preserved across different lineages

**Fig. 4:**
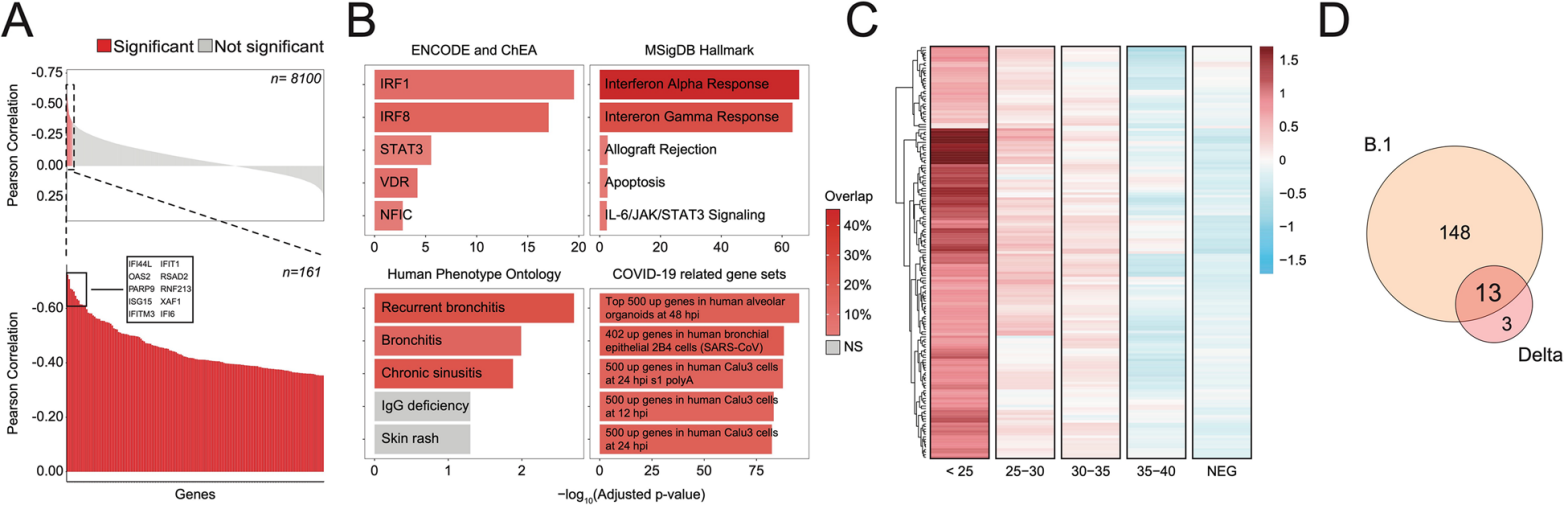
Transcriptional profiling of SARS-CoV-2 infected patients reveals a gene signature correlated with viral load and preserved across different lineages. **A** Correlation analysis between CTs and gene expression of B.1 patients, performed on 8100 genes, is shown as a barplot. For each gene (*x*-axis), its correlation value (y-axis) and significance (*p*-value < 0.0001, red) is reported. Bottom: highlight of the significant results. (161 genes). The top 10 most anti-correlated genes are reported (black box). **B** Pathway and gene set enrichment analysis performed for different databases using the gene signature previously identified. Each barplot shows the significance (*x*-axis) and the percentage of overlap (fill color) between the input signature and the tested public genesets. **C** Heatmap of *z*-scored, log2-transformed, and normalized gene counts for the 161 significantly correlated genes from **A**. Values have been averaged in 4 groups of samples depending on the CT (*x*-axis) or whether they were negative. **D** Venn diagram of significantly anti-correlated genes between B.1 (161 genes) and Delta (16 genes) variant-infected patients

The comprehensive gene expression profiling of the respiratory epithelium of patients positive for SARS-CoV-2 infection holds great promises in terms of preventive, diagnostic, and therapeutic advancements. For this reason, we implemented an RNA-seq workflow adapted to work with diagnostic swabs, known to have extremely low quantity and quality of RNA. We processed around 700 samples, divided in two batches for the analysis of the differential molecular host response to B.1 and Delta variants infection (Additional file 1: Fig. S4A). After filtering, the B.1 final dataset comprehended 116 SARS-COV-2 positive samples to be compared with 88 negative ones. On the other hand, the Delta dataset was composed of 43 and 95 SARS-COV-2 positive and negative samples, respectively (Additional file 1: Fig. S4A). Although the cohort of patients was numerous, in both cases many confounding variables influenced the possibility to compare positive and negative conditions. Inter-patient heterogeneity, different viral loads, and swab-related variability are some of the factors that prevented us from finding a strong variance solely related to the presence or absence of the infection (Additional file 1: Fig. S4B). Therefore, we decided to take advantage, again, of the Ct values associated with positive samples and perform a correlation analysis between gene expression and viral load, starting with the B.1 dataset (Fig. 4A top). After filtering non-expressed genes (see Methods), a Pearson correlation test was conducted and a signature of 161 genes (Additional file 2: Table S7) was found to be significantly anti-correlated with Ct values (*p*-value < 0.0001, Fig. 4A bottom). Among the 10 most anti-correlated genes, many downstream targets of interferon antiviral response (e.g., IFI44L, OAS2, PARP9, IFITM3, IFIT1) were found, as already reported from in vitro experiments and single-cell studies [32]. We confirmed an enhanced antiviral immune response by performing pathway and gene signatures enrichment analyses (Fig. 4B). Indeed, together with COVID-19- and Bronchitis-related signatures, the most significant results comprehended Interferon Alpha pathway and its inducers, IRFs. Additionally, STAT3-regulated genes were enriched, which were recently found to be aberrantly activated upon SARS-CoV-2 infection [33] (Fig. 4B). Interestingly, when looking at the expression levels of these genes in our cohort, negative patients displayed a transcriptional behavior comparable to samples with the lowest viral load (Fig. 4C). We applied the same approach to the Delta dataset and retrieved a molecular signature of 16 genes (Fig. 4D and Additional file 1: Fig. S4C-E), way smaller than the other one, most probably due to the restricted number of patients. Nevertheless, almost every gene (13 genes, 81.25%) was common to the B.1 signature and belonged to the same pathways (IFIT3, OAS3, IFI6 - Fig. 4D). With that said, our CT-based approach overcomes all the technical and biological variability related to the direct use of regular swabs extracts and establishes a robust gene signature that is preserved across different viral lineages and could be used as biomarkers for disease monitoring, prevention, and non-conventional treatments.

## Discussion

Genomic surveillance using Next Generation Sequencing approaches has proven its extreme efficacy in some of the most notorious outbreaks of the 21st century [34–36]. The technology, indeed, allows to specifically identify the pathogen genome variability directly from clinical specimens, not relying on the traditional and time-consuming isolation and in vitro cultivation steps. As further proof of its potential, genome sequencing is now considered the standard genotyping procedure for *influenza* virus and has already been used for decision-making in terms of vaccine development by CDC [37]. Similarly, with the emergence of SARS-CoV-2 and its rapid evolution towards more and more infective variants [3, 4] genomic surveillance had a critical role in monitoring virus evolution and detecting new mutations. Nevertheless, several countries still lack an efficient or homogenous integrated program for SARS-CoV-2 genome sequencing [38]. Such observation depends on several factors in a country-specific manner. The amount of resources NGS technologies rely on is probably an important factor for emerging countries. The approach we optimized aims at generating an affordable and easy program that can be translated to monitoring viral variability at the regional level; a strategy that could allow emerging economies to perform efficient surveillance. This approach does not rely on any specific automation and can be implemented by a three-person team on any short-read sequencer. We proved its applicability to as few as 1.25 μL of unquantified RNA, enabling us to scale down the library reaction volumes, and thus the costs, while not affecting the sequencing metrics. The percentage of consensus sequences retrieved in each sequencing is only slightly affected when pooling up to 384 samples per flow cell, corresponding to 5 million reads per sample. Similar results were obtained when simulating 2.5 million reads per sample, thus demonstrating that, in principle, it is possible to sequence up to 1536 samples per run when using two flow cells in parallel.

We also tested a fast protocol which, by merging two PCR steps in one step, allows to speed up the library generation times by 40%. While in this case, the general sequencing quality is lower, the solution still enables retrieving a consensus sequence for about 60% of the analyzed samples. Such a number is still enough for screening purposes to identify the main circulating variants and might be applied when time is a critical factor, as during major infection waves. It indeed allows to easily sequence over 4000 samples in one month, allowing the detection of variants with a frequency of about 1% and comply with EC/ECDC recommendation to sequence at least 5% of all positive cases.

As proof of principle, we applied the framework to the 2021 genomic surveillance of the most densely populated region in Italy, Campania. As a result, the region is now the one with the highest number of sequences deposited on GISAID.

One of the biggest limitations of lacking microbiological surveillance relies on the inability to detect and isolate new emerging variants, increasing the chances of new waves of infections. The most adopted evolutionary model to study SARS-CoV-2 relies on the assumption that each of the lineages spread from an original ancestor originated in a given space and time. Being able to deeply profile the pandemic’s dynamics at a regional level, is thus critical for the detection of such ancestors as soon as they start to emerge and spread. We demonstrated that, even looking at restricted territory, as Campania is, new mutations can be identified. In addition, we actually observed several lineages potentially originating in Campania, including VOC sub-variants (as the P.1.1, BA.1.21.1) that spread worldwide. Some of these variants were designated by the pangolin system only after our alert. We indeed propose three different principles to investigate and, potentially, define novel lineages. First, evaluate the presence of known pathogenic SNPs in unexpected lineages. Second, observe the co-occurrence of mutations with increasing frequency over time. Finally, look at the emergence of new mutations in long hospitalized infections. The latter approach emphasizes the synergy between the healthcare centers, which provide clinical metadata, and the sequencing facilities that generate the viral consensus sequences. Thanks to these approaches we were able to discover and describe three new lineages (B.1.177.88, Q.2, BA.1.21.1), and helped to guide local policy-makers in the establishment of localized containment areas in the Region. For instance, the “Agro Nocerino-Sarnese” area was quarantined after pointing out the emergence of the B.1.177.88 variant; this decision likely prevented the spreading of the variant and it disappeared a few weeks later.

Among the others, we also collected over time several samples from patients with persistent SARS-CoV-2 infections. Interestingly none of the patients completed a full SARS-CoV-2 vaccination cycle and the vast majority had no vaccination at all. In only one patient out of 20, we were able to actually detect the rising of a new mutation (in viral NTP/helicase NSP13 R339C), in the viral genome. The identification of this mutation can be associated to two possible events: (i) the virus actually acquired a new mutation in the host or, less likely but possible, (ii) the mutation pre-existed at low frequencies as part of the quasispecies infecting the host and was then fixed in the viral population. It is worth noting that the mutation identified is extremely rare worldwide and it was identified on GISAID only 23 times in the same variant of the patient under investigation (*alpha*). However, while this observation alone does not necessarily imply the identification of a new lineage, it strongly suggests that viral populations in patients with persistent infections can potentially evolve.

In conclusion, in this study, we propose a cost-effective and rapid workflow for SARS-CoV-2 genome sequencing whose cost per sample is 5 times lower than the standard application for SARS-CoV-2 WGS (using solution B). Moreover, our approach is based on PCR enrichment and amplification of viral genomes, thus not requiring any specialized skill and suitable to be performed after a minimum training. Finally, the possibility to pool 384 samples or more in each sequencing flow cell, allows a 3-person team (two wet scientists and one bioinformatician) to deliver the sequences of over 760 samples in as few as 6 days (with ~ 5h of hands-on time). Taken all together, these properties make our approach not only highly valuable in monitoring COVID-19 pandemics, as we showed at a regional level, but also easily transferable to other genomic centers.

Main limitation of the approach is its amplicon-based nature, which requires the monitoring of the primers used as the viral genome mutates over time. Nevertheless, other strategies used for SARS-CoV-2 genome sequencing, e.g., probe-based enrichment and metagenome WGS, are either more time-consuming and expensive (probe-based enrichment) or deeply affected by host and microbial genetic material (metagenome). In addition, the use of short-reads, as for all second-generation sequencing strategies, has a potential impact on the capability to discern viral recombination from patient co-infection, the former being the main feature in coronaviruses [39]. While such an issue can not be solved without a long-read approach, we argue that any possible spread of recombinant strains would be recognized by the co-occurence of the same mutations associated to different variants in several samples, as proven by the detection of two XA recombinant variants in our dataset.

The general lack of bioinformatics skills required for raw data analysis is a critical factor for NGS technologies implementation in clinical diagnostic laboratories. While offering a simple and cheap approach for SARS-CoV-2 genome sequencing, our workflow also relies on the use of bioinformatics tools for data interpretation. We addressed this problem by developing a comprehensive pipeline which requires minimum informatics skills. Once started, the pipeline performs all the analysis required for the production of the consensus sequence and automatically performs the upload of high-quality sequences to GISAID.

Eventually, we identified molecular signatures from COVID-19 patients’ gene expression that agree with identified biomarkers reported in previous studies. Our approach extends the scope of SARS-CoV-2 genomic surveillance, as it allows for the examination of in vivo samples characterized by the predominance of degraded RNA molecules. This competence enables overcoming the limitation of in-vitro and single-cell studies, such as model-specific variations and a small number of samples limit, respectively. Gene expression data from COVID-19 patients might have a pivotal role as a bridge between genomic data and translational medicine. On one hand, finding a gene signature that describes and defines the patient status after SARS-CoV-2 infection may be useful to understand the pathogenesis of the virus in different patients and patients’ status. On the other hand, it might be used to evaluate new therapeutic treatments. In this study, we propose a cost-effective and rapid workflow to produce these data and to retrieve biologically relevant biomarkers. Furthermore, the RNA-seq analysis implemented in our workflow offers for the first time a comparison between molecular signatures from RNAs of different SARS-COV-2 variants, proving that the transcriptional host response of the upper airways changes in the same direction, regardless of the viral variant they have been infected by. We also envision integrating this approach with other types of metadata (e.g., patient symptomatology) to achieve the aforementioned goals.

## Conclusions

Here we developed a fast and cost-effective approach for SARS-CoV-2 genomic surveillance. The proposed strategy allows to scale of viral genome sequencing down to 10 times less per sample. In addition, this protocol minimizes the hands-on time and does not require intensive training or any particular automation. Taken altogether, these features allowed us to profile the SARS-CoV-2 pandemic in Campania (Italy) during 2020-2021. We thus identified the main variants leading each infection wave in the regional territory and discovered 3 new SARS-CoV-2 lineages specifically originated in Campania, demonstrating the potential of genomic surveillance. We also added a further layer of information by integrating viral genotype with host upper respiratory airways transcriptome upon infection. This integrative point of view revealed a gene-expression signature correlated with viral loads and characterizing real-world infected patients. Finally, we showed that the host airways epithelium response to SARS-CoV-2 infection is not significantly different in B.1 and *delta* variant infected patients. In conclusion, we believe that the proposed approach can significantly help fight against the pandemic by democratizing viral genome profiling through next-generation sequencing.

## Supplementary Information

**Additional file 1: Figure S1.** A) Boxplots showing the percentage of samples with 100x genome coverage (left), million reads produced (centre) and mapped (right), divided by each tested solution. Only samples with average Ct value < 33 were considered. **Figure S2.** A) Histogram representing the cohort of patients of this study. Age (x axis) and sex (colors) are reported. B) Barcharts showing the distribution, in time, of the samples assigned to variants of concern and of interest identified in the study. C) Distribution of Ct values, in time, for all the samples collected during the pandemic in Campania. The trend line (red) and 95% confidence interval (light gray) are shown. **Figure S3.** A) Donut charts representing the amount of analyzed genomes presenting some mutation of concern, namely Spike L18F, S477N and P681H, divided by lineage. The definition of Expected lineage is described in the Methods. B) Phylogeny of the proposed lineage from Fig. 3A, the proposed lineage is in green. Bootstrap values for each node are shown as node points. C) Results from the clustering analysis for samples collected until May 2022, displayed as line plots of frequency over time (trends). The arrow indicates the investigated cluster in Fig. 3D. D) Phylogeny of the proposed lineage from Fig. 3D, the proposed lineage is in green. Bootstrap values for each node are shown as node points. E) Results from the clustering analysis for samples collected until January 2022, displayed as line plots of frequency over time (trends). The arrow indicates the investigated cluster F) Phylogeny of the proposed BA.1 sublineage, the proposed new variant is in green. Bootstrap values for each node are shown as node points. **Figure S4.** A) Schematic representation of RNA-seq data structure, pre- and post-filtering. B) Principal Component Analysis plots of B.1 and Delta datasets, colored by SARS-COV-2 infection positivity. C) Correlation analysis between CTs and gene expression of Delta patients, performed on 5525 genes, is shown as a barplot. For each gene (x axis), its correlation value (y axis) and significance (*p*-value < 0.0001, red) is reported. Bottom: highlight of the significant results. (16 genes). D) Pathway and gene set enrichment analysis performed for different databases using the gene signature previously identified. Each barplot shows the significance (x axis) and the percentage of overlap (fill color) between the input signature and the tested public genesets. E) Heatmap of z-scored, log2-transformed and normalized gene counts for the 16 significantly correlated genes from the analysis of Delta dataset. Values have been averaged in 3 groups of samples depending on the CT (x axis) or whether they were negative.

**Additional file 2: Supplementary tables 1-6**. Extended table 1: Cost details for each solution. Extended table 2: Summary of all data produced. Extended table 3: List of the 6970 mutations found in this study, sorted by position along the Sars-CoV-2 genome. Extended table 4: List of the 194 mutations first detected in Campania, sorted by position along the Sars-CoV-2 genome. Extended table 5: List of lineages first detected in Campania. Extended table 6: World distribution of lineages first detected in Campania. Extended table 7: List of 161 genes correlated with viral load.

**Additional file 3.** Laboratory protocol adopted in this work for SARS-CoV-2 WGS library generation (“Solution B”).

## Data Availability

All SARS-CoV-2 sequencing data are available through the GISAID database. All analyses were performed by exporting sample metadata and PANGO lineage from GISAID (at the date of 2022-03-22, GISAID accession number: EPI_SET_20220718pm)), by including only full genomes and excluding those at low coverage as described above. RNA-seq gene expression data are available at GEO Datasets (GSE184610). Data analysis pipeline is freely available for non-commercial use upon the signature of an institutional MTA at: https://gitlab.com/nextgd/ngdx-atoplex-panel-covid-19-pipeline. The laboratory protocol of our approach (solution B) is provided in Additional file 3.
