# Supplementary material for: Improved SARS-CoV-2 sequencing surveillance allows the identification of new variants and signatures in infected patients
Source: Genome Med. 2022 Aug 12;14:90. doi: 10.1186/s13073-022-01098-8 (PMC9372932; doi:10.1186/s13073-022-01098-8)
Supplement: Supplementary file 1 — Additional file 1: Figure S1. A) Boxplots showing the percentage of samples with 100x genome coverage (left), million reads produced (centre) and mapped (right), divided by each tested solution. Only samples with average Ct value < 33 were considered. Figure S2. A) Histogram representing the cohort of patients of this study. Age (x axis) and sex (colors) are reported. B) Barcharts showing the distribution, in time, of the samples assigned to variants of concern and of interest identified in the study. C) Distribution of Ct values, in time, for all the samples collected during the pandemic in Campania. The trend line (red) and 95% confidence interval (light gray) are shown. Figure S3. A) Donut charts representing the amount of analyzed genomes presenting some mutation of concern, namely Spike L18F, S477N and P681H, divided by lineage. The definition of Expected lineage is described in the Methods. B) Phylogeny of the proposed lineage from Fig. 3A, the proposed lineage is in green. Bootstrap values for each node are shown as node points. C) Results from the clustering analysis for samples collected until May 2022, displayed as line plots of frequency over time (trends). The arrow indicates the investigated cluster in Fig. 3D. D) Phylogeny of the proposed lineage from Fig. 3D, the proposed lineage is in green. Bootstrap values for each node are shown as node points. E) Results from the clustering analysis for samples collected until January 2022, displayed as line plots of frequency over time (trends). The arrow indicates the investigated cluster F) Phylogeny of the proposed BA.1 sublineage, the proposed new variant is in green. Bootstrap values for each node are shown as node points. Figure S4. A) Schematic representation of RNA-seq data structure, pre- and post-filtering. B) Principal Component Analysis plots of B.1 and Delta datasets, colored by SARS-COV-2 infection positivity. C) Correlation analysis between CTs and gene expression of Delta patients, performed on 5 [file 13073_2022_1098_MOESM1_ESM.pdf]

A

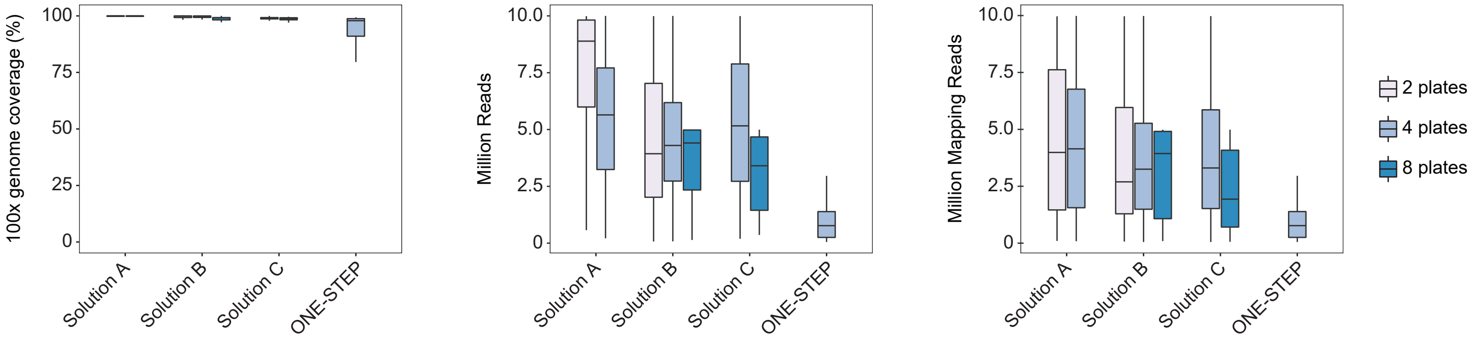

Figure S1. A) Boxplots showing the percentage of samples with 100x genome coverage (left), million reads produced (centre) and mapped (right), divided by each tested solution. Only samples with average Ct value < 33 were considered.

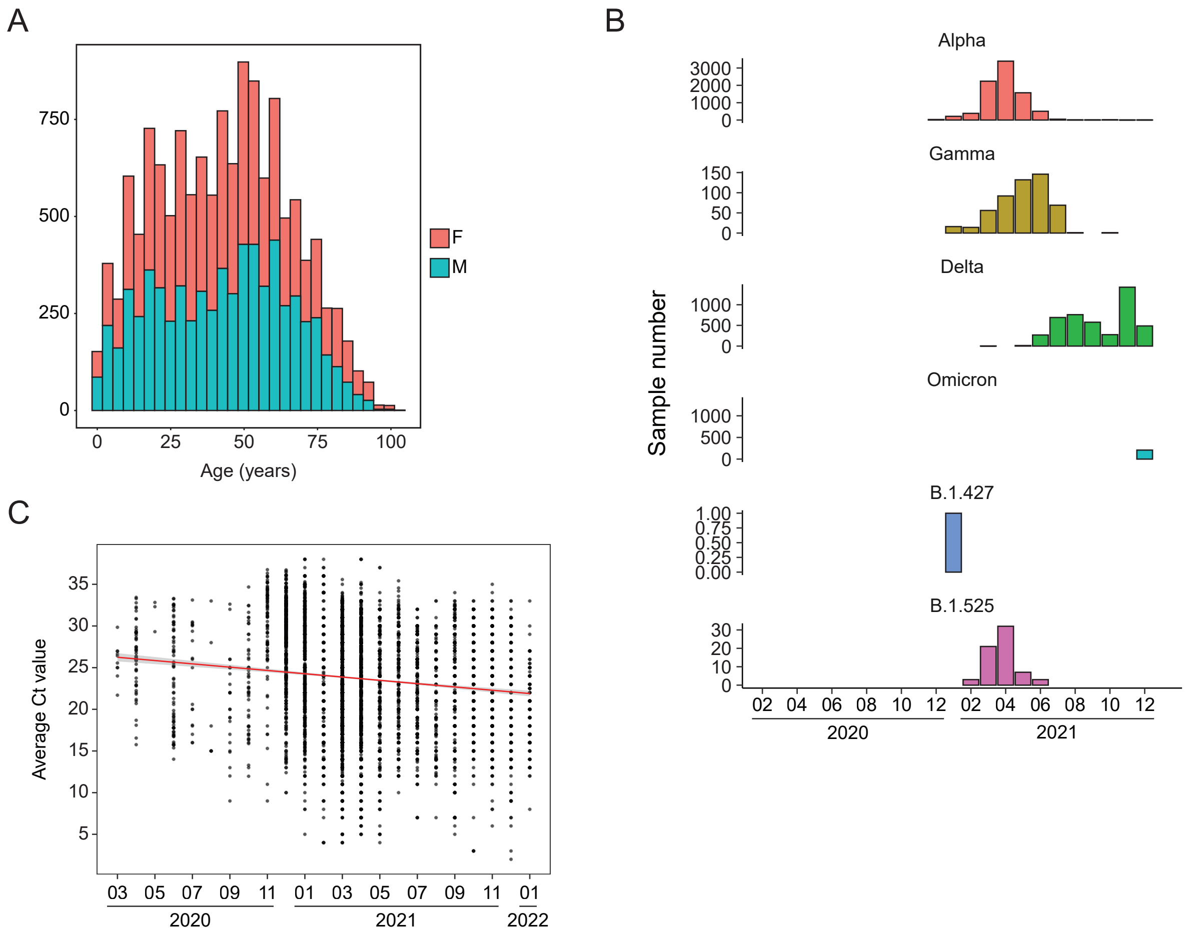

Figure S2. A) Histogram representing the cohort of patients of this study. Age (x axis) and sex (colors) are reported. B) Barcharts showing the distribution, in time, of the samples assigned to variants of concern and of interest identified in the study. C) Distribution of Ct values, in time, for all the samples collected during the pandemic in Campania. The trend line (red) and 95% confidence interval (light gray) are shown.

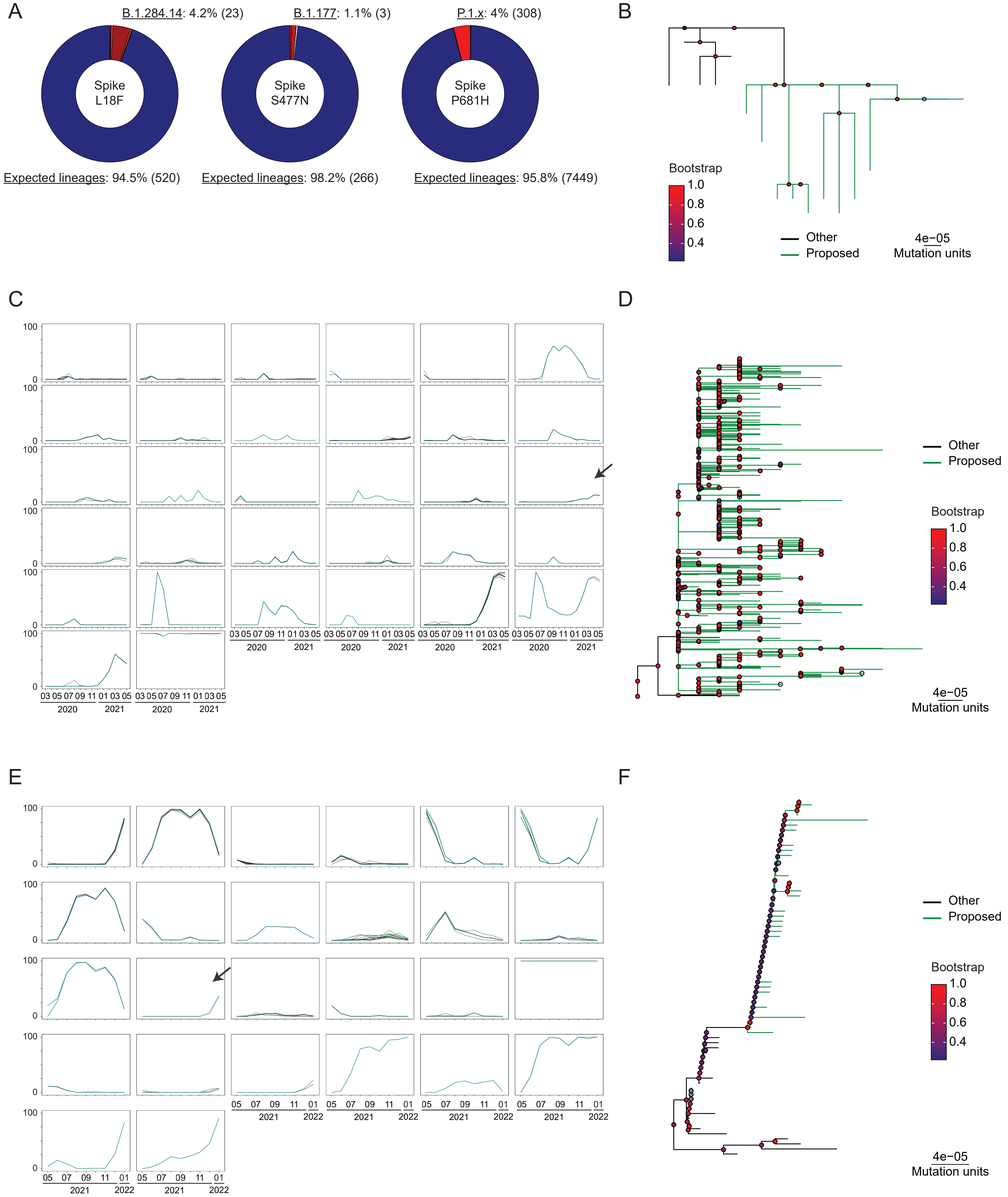

Figure S3. A) Donut charts representing the amount of analyzed genomes presenting some mutation of concern, namely Spike L18F, S477N and P681H, divided by lineage. The definition of Expected lineage is described in the Methods. B) Phylogeny of the proposed lineage from Fig. 3A, the proposed lineage is in green. Bootstrap values for each node are shown as node points. C) Results from the clustering analysis for samples collected until May 2022, displayed as line plots of frequency over time (trends). The arrow indicates the investigated cluster in Fig. 3D. D) Phylogeny of the proposed lineage from Fig. 3D, the proposed lineage is in green. Bootstrap values for each node are shown as node points. E) Results from the clustering analysis for samples collected until January 2022, displayed as line plots of frequency over time (trends). The arrow indicates the investigated cluster F) Phylogeny of the proposed BA.1 sublineage, the proposed new variant is in green. Bootstrap values for each node are shown as node points.

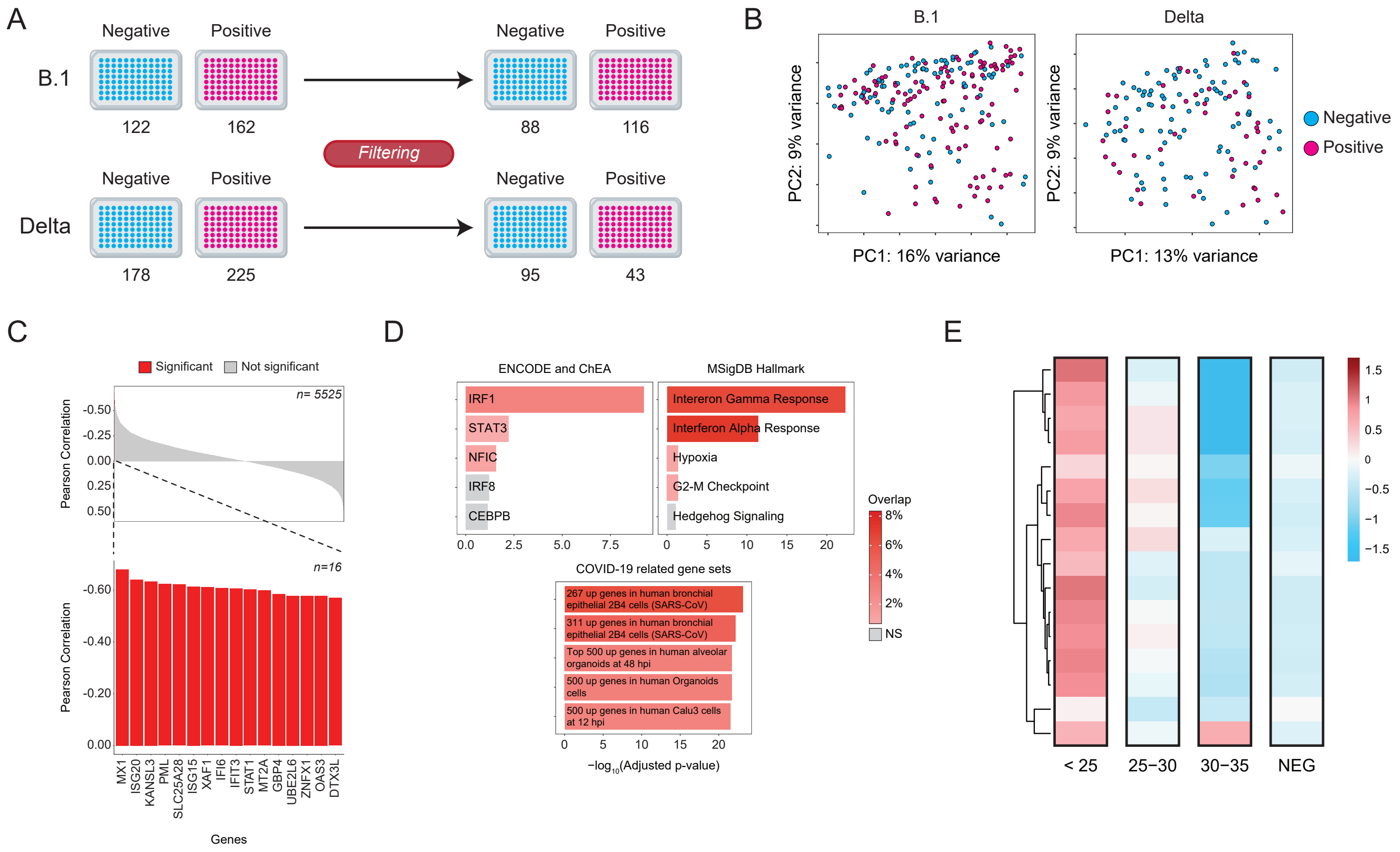

Figure S4. A) Schematic representation of RNA-seq data structure, pre- and post-filtering. B) Principal Component Analysis plots of B.1 and Delta datasets, colored by SARS-COV-2 infection positivity. C) Correlation analysis between CTs and gene expression of Delta patients, performed on 5525 genes, is shown as a barplot. For each gene (x axis), its correlation value (y axis) and significance (p-value < 0.0001, red) is reported. Bottom: highlight of the significant results. (16 genes). D) Pathway and gene set enrichment analysis performed for different databases using the gene signature previously identified. Each barplot shows the significance (x axis) and the percentage of overlap (fill color) between the input signature and the tested public genesets. E) Heatmap of z-scored, log2-transformed and normalized gene counts for the 16 significantly correlated genes from the analysis of Delta dataset. Values have been averaged in 3 groups of samples depending on the CT (x axis) or whether they were negative.
