# Supplementary material for: Improved SARS-CoV-2 sequencing surveillance allows the identification of new variants and signatures in infected patients
Source: Genome Med. 2022 Aug 12;14:90. doi: 10.1186/s13073-022-01098-8 (PMC9372932; doi:10.1186/s13073-022-01098-8)
Supplement: Supplementary file 3 — Additional file 3. Laboratory protocol adopted in this work for SARS-CoV-2 WGS library generation (“Solution B”). [file 13073_2022_1098_MOESM3_ESM.docx]

**SARS-CoV-2 sequencing library (solution B)**

The following protocol allows generating MGI DNBseq compatible libraries for SARS-CoV-2 genotyping. It is based on the “MGI Atoplex RNA Universal Library Preparation Module” user manual.

**NOTE:** To avoid aerosol contamination of PCR products, the following procedures are to be performed in three separate working areas and PCR hoods. Use dedicated gloves and coats in each area and **perform cleaning with** DNA Zap (Thermofisher scientific Cat. No. AM9890) before and after each procedure.
For every 96 libraries to generate, we suggest preparing a mix for 104 samples.

**Protocol:**

1. **Reverse transcription**
   1. **In the first dedicated area**, spot 2.5 µL of each sample RNA (total RNA extracted from throat swabs. No quantification is needed) into a 96 well.
   2. Add 2.5 µL RTmix (table below) to each sample:

| **Components** | **Volume (µL) (1 sample)** | **Volume (µL) (104 samples)** |
| --- | --- | --- |
| N6 Buffer | 1 | 104 |
| RT Buffer | 1.25 | 130 |
| RT Enzyme mix | 0.25 | 26 |
| **Total** | **2.5** | **260** |

- 1. Put the samples into the thermocycler and run the following program (~ 55 min):

| Lid | 105 °C |
| --- | --- |
| **Temperature (°C)** | **Time (min)** |
| 25 | 10 |
| 42 | 30 |
| 70 | 15 |
| 4 | hold |

1. **1^st^ PCR amplification and SARS-CoV-2 genome enrichment**
   1. Prepare the 1st PCR Amplification Mixture (table below) and add 7.375 µL mix to each sample.

| **Components** | **Volume (µL) (1 sample)** | **Volume (µL) (104 samples)** |
| --- | --- | --- |
| PCR Enzyme Mix | 6.25 | 650 |
| PCR Clean Enzyme | 0.125 | 13 |
| PCR Primer Pool | 1 | 104 |
| **Total** | **7.375** | **767** |

- 1. Put the samples into the thermocycler and run the following program (~ 1h):

| Lid | 105 °C |  |
| --- | --- | --- |
| **Temperature (°C)** | **Time** | **Cycles** |
| 37 | 5 min | 1 |
| 95 | 10 min |  |
| 95 | 15 s | 13 |
| 64 | 1 min |  |
| 60 | 1 min |  |
| 72 | 30 s |  |
| 4 | hold |  |

1. **1^st^ PCR cleanup**
   1. **In the second dedicated area**, add 15 µL of magnetic beads into each sample and mix 10 times.
   2. Incubate at RT for 5 minutes.
   3. Put the 96 well on a magnetic stand to allow the separation of beads from the solution.
   4. Remove the supernatant. Avoid touching the beads pellet.
   5. Add 150 µL of freshly prepared Ethanol 80%, wait for 30 sec and discard the wash.
   6. Repeat the previous step and pay attention to remove all the ethanol.
   7. Air-dry the beads at RT for about 2 minutes, until no reflectiveness is observed.
   8. Remove the plate from the magnetic stand and resuspend the beads in 3.5 µL Elution Buffer.
      After purification, the samples can be stored overnight at -20°C.
2. **2^nd^ PCR amplification**
   1. Prepare the 2nd PCR Amplification Mix and add 7.125 µL of it to each sample:

| **Components** | **Volume (µL) (1 sample)** | **Volume (µL) (104 samples)** |
| --- | --- | --- |
| PCR Enzyme Mix | 6.25 | 650 |
| PCR Clean Enzyme | 0.125 | 13 |
| PCR Additive | 0.25 | 26 |
| PCR Block | 0.5 | 52 |
| **Total** | **7.125** | **741** |

- 1. Add 2 µL PCR Dual Barcode Primer Mix (01-96) by choosing a different barcode for each sample.
  2. Put the samples into the thermocycler and run the following program (~ 2h):

| Lid | 105 °C |  |
| --- | --- | --- |
| **Temperature (°C)** | **Time** | **Cycles** |
| 37 | 5 min | 1 |
| 95 | 10 min |  |
| 95 | 15 s | 27 |
| 64 | 1 min |  |
| 60 | 1 min |  |
| 72 | 30 s |  |
| 4 | hold |  |

**2^nd^ PCR clean up**

- 1. **In the thirds dedicated area**, add 11 µL of magnetic beads into each sample and mix 10 times.
  2. Repeat steps from 3.2 to 3.7
  3. Remove the plate from the magnetic stand and resuspend the beads in a 12 µL Elution Buffer.
  4. Incubate at RT for 5 minutes, then put back on the magnetic stand and collect the supernatant.

1. **Libraries quantification, quality control and sequencing**
   1. Quantify each sample using Qubit dsDNA HS Assay Kit.
   2. Prepare an equimolar pool for each 96 well and quantify it. The concentration of the pool should be at least 8,3 ng/ µL and the final volume at least 48 µL.
   3. Use 400 ng DNA from each pool as input for circularization. Follow MGI guidelines.
   4. Each circularized pool can be loaded on a separate lane of an MGI DNBSEQ-G400 sequencer.
